# Low Density Granulocytes in ANCA Vasculitis Are Heterogenous and Hypo-Responsive to Anti-Myeloperoxidase Antibodies

**DOI:** 10.3389/fimmu.2019.02603

**Published:** 2019-11-07

**Authors:** Aisling Ui Mhaonaigh, Alice M. Coughlan, Amrita Dwivedi, Jack Hartnett, Joana Cabral, Barry Moran, Kiva Brennan, Sarah L. Doyle, Katherine Hughes, Rosemary Lucey, Achilleas Floudas, Ursula Fearon, Susan McGrath, Sarah Cormican, Aine De Bhailis, Eleanor J. Molloy, Gareth Brady, Mark A. Little

**Affiliations:** ^1^Trinity Health Kidney Centre, Trinity Translational Medicine Institute, Trinity College Dublin, Dublin, Ireland; ^2^Department of Clinical Medicine, School of Medicine, Trinity College Dublin, Dublin, Ireland; ^3^The Regenerative Medicine Institute (REMEDI), National University of Ireland, Galway, Ireland; ^4^School of Biochemistry and Immunology, Trinity Biomedical Sciences Institute, Trinity College Dublin, Dublin, Ireland; ^5^National Children's Research Centre, Our Lady's Children's Hospital Crumlin, Dublin, Ireland; ^6^Molecular Rheumatology, Trinity Biomedical Sciences Institute, Trinity College Dublin, Dublin, Ireland; ^7^Department of Paediatrics, Trinity Translational Medicine Institute, Trinity College Dublin, Dublin, Ireland; ^8^Irish Centre for Vascular Biology, Trinity College Dublin, Dublin, Ireland

**Keywords:** ANCA associated vasculitis, low density granulocytes, anti-MPO, reactive oxygen species, neutrophil heterogeneity

## Abstract

Low Density Granulocytes (LDGs), which appear in the peripheral blood mononuclear cell layer of density-separated blood, are seen in cancer, sepsis, autoimmunity, and pregnancy. Their significance in ANCA vasculitis (AAV) is little understood. As these cells bear the autoantigens associated with this condition and have been found to undergo spontaneous NETosis in other diseases, we hypothesized that they were key drivers of vascular inflammation. We found that LDGs comprise a 3-fold higher fraction of total granulocytes in active vs. remission AAV and disease controls. They are heterogeneous, split between cells displaying mature (75%), and immature (25%) phenotypes. Surprisingly, LDGs (unlike normal density granulocytes) are hyporesponsive to anti-myeloperoxidase antibody stimulation, despite expressing myeloperoxidase on their surface. They are characterized by reduced CD16, CD88, and CD10 expression, higher LOX-1 expression and immature nuclear morphology. Reduced CD16 expression is like that observed in the LDG population in umbilical cord blood and in granulocytes of humanized mice treated with G-CSF. LDGs in AAV are thus a mixed population of mature and immature neutrophils. Their poor response to anti-MPO stimulation suggests that, rather than being a primary driver of AAV pathogenesis, LDGs display characteristics consistent with generic emergency granulopoiesis responders in the context of acute inflammation.

## Introduction

Neutrophils have conventionally been considered a uniform, short-lived, and functionally-restricted population of immune cells (1). Recent evidence suggests that they feature a plasticity that allows them to respond and adapt to different disease situations (2, 3). Anti-neutrophil cytoplasm autoantibody (ANCA) vasculitis (AAV) is a systemic autoimmune disease in which neutrophils play a pivotal role (4). It is characterized by autoantibodies directed against neutrophil proteins myeloperoxidase (MPO) and proteinase-3 (PR3) and is associated clinically with rapidly progressive glomerulonephritis and inflammatory necrosis of small blood vessels in lungs, skin, and other organs (5–7). Neutrophils obtained from patients with active AAV aberrantly transcribe the autoantigens MPO and PR3, a feature that correlates with subsequent clinical outcome (8).

Low density granulocytes (LDGs) are distinct from normal density granulocytes (NDGs) with a density below 1.07 g/ml and sediment in the PBMC layer after density gradient fractionation of whole blood (3, 9, 10). LDGs are expanded in animal models of viral-infection (11) and arthritis (12) and in humans with cancer (13), sepsis (14), HIV (9, 15) and various autoimmune conditions, including systemic lupus erythematosus (SLE) (3), rheumatoid arthritis (RA) (16), and psoriasis (17). There is lack of clarity in the phenotypical and functional characteristics of LDGs, and in the relationship of LDGs to myeloid-derived suppressor cells (MDSCs) (Table 1). Most studies in autoimmune diseases suggest that LDGs are pro-inflammatory, relatively long-lived and undergo NETosis more readily than NDGs (3, 23). Therefore, it has been postulated that these cells are a key pathogenic force of autoimmunity (25).

**Table 1:** LDG population characteristics in various disease conditions.

**Disease area**	**Specific condition**	**Population**	**Surface marker expression**	**Arginase**	**Nuclear Morphology**	**ROS**	**Reference**
Infection	HIV	LDGs	CD15^+^,CD11b^+^,CD13^+^,CD33^+^, CD16^Int/lo^,CD66b^+^CD63^+^	Decreased	Mature		(9)
	TB	LDGs	CD15^+^,CD14^low^ CD16^+^, CD33^+^, CD66b^+^ and CD62L^low^,		Mature	Increased in LDGs	(18)
	Sepsis	Interphase neutrophils	CD16^int^, CD11b^+^, CD15^+^, CD33^−/low^, CD54^−/low^, CD62L^−/low^, CD66b^+^ and CD14^−/low^ HLA-DR^−/low^	Increased	Heterogeneous mixed banded and segmented	ND	(14)
Malignancy	Cancer	LDGs	CD66b^+^, CD33^+^, CD16^var^, CD11b^Var^, CD125^−^ HLA-DR^−^		Immature		(19)
	Cancer	G-MDSC	CD11b^+^, CD14^−^,CD15^+^, CD66b^+^, LOX-1^var^	Increased expression in Lox-1^+^	Lox-1^+^ mature, Lox-1^−^ immature	LOX-1^+^ increased	(20)
	Hepatocellular carcinoma	G-MDSC	CD11b^+^,CD14^−^,HLA-DR^−/low^,CD15^+^, LOX-1^+^	Increased on CD15+ Lox-1+	ND	Increased	(21)
	Renal cell carcinoma	MDSC	CD66b^+^,CD11b^+^,VEGFR1^+^, CD62l ^low^,CD16^low^	Decreased	Heterogeneous, 90% segmented	ND	(22)
Autoimmunity	Rheumatoid arthritis	LDGs	CD10^+^,CD14^+^,CD15^+^ CD16^int/low^	ND	ND	Lower than NDGs	(16)
	Psoriasis	LDGs	CD10^+^CD14^low^	ND	ND		(17)
	SLE	LDGs	CD10^+^,CD11c^lo^,CD14^lo^, CD15^hi^,CD16^hi^, CD31^+^, CD114^+^, CD116^−^	ND^*^	Heterogeneous, Mature, less segmented	ND	(3, 23)
Other	G-CSF treated donors	LDNs	CD66b^+^,CD11b^var^,CD10^var^, CD16^var^	Increased mRNA, decreased activity	Heterogeneous mixed banded and segmented	Not involved in T Cell suppression	(24)
	Pregnancy	LDGs	CD15^+^, CD66b^+^,CD63^+^,CD33^+^, CD16^int/low^	Increased on cord vs maternal	ND	ND	(10)

* *HIV, Human Immunodeficiency Virus; TB, Tuberculosis; LDG, Low Density Granulocytes; G-MDSC, Granulocytic Myeloid Derived Suppressor Cells; LDN, Low Density Neutrophils; SLE, Systemic Lupus Erythematosus; NDG, Normal Density Granulocytes; ND, Not determined; LOX-1, Lectin-type Oxidized LDL receptor-1; var, variable; G-CSF, Granulocyte Colony Stimulating factor*.

Traditional flow cytometric markers to identify neutrophil populations within highly granulated populations include CD66b, CD15, CD11b, and CD16. However, the expression of these surface receptors can be altered upon neutrophil activation and following density centrifugation (26, 27). Despite recent work to consolidate phenotypic description of these cells, many different ways of identifying LDGs are present in the literature (28). Potentially useful distinguishing markers include CD10, which distinguishes mature from immature neutrophils, and lectin-type oxidized LDL receptor 1 (LOX-1) (20, 24).

To investigate the role of LDGs in AAV, we combined traditional and imaging flow cytometric analysis with functional assays. We found that, in active AAV, the LDG population is expanded and comprised of a heterogeneous population of neutrophils, with differential expression of CD16 and CD10. A substantial fraction of LDGs are immature neutrophils, likely released in response to emergency granulopoiesis. We found that, unlike NDGs, LDGs are hyporesponsive to stimulation with monoclonal antibodies directed against MPO, suggesting that they may not have an important pathogenic role in AAV.

## Materials and Methods

### Patients

We recruited AAV patients with acute disease (*n* = 13), those in remission (*n* = 6), age matched healthy controls (HC, *n* = 5) and disease controls (DC, a mix of renal impairment and non-AAV systemic inflammation, *n* = 11, Chronic kidney disease *n* = 3, Coronary artery disease *n* = 1, Stroke *n* = 1, Colorectal carcinoma *n* = 1, IgA vasculitis *n* = 1, rheumatoid arthritis *n* = 4) (Table 2). All patients with AAV fulfilled the revised Chapel Hill Consensus Conference classification (29). Active AAV was defined by a Birmingham vasculitis activity score (BVAS) ≥2 and remission by BVAS = 0. Disease/healthy controls and patients with AAV were recruited from the Rare Kidney Disease Registry and Biobank (www.tcd.ie/medicine/thkc/research/rare.php). Umbilical cord blood (UCB) was obtained from mothers undergoing vaginal deliveries with healthy term pregnancy; the babies had normal Apgar scores. The study was approved by institutional ethics committees of Tallaght, St Vincent's, St James and Beaumont Hospitals, and all recruits provided written informed consent.

**Table 2:** Baseline characteristics of the study subjects, by disease classification.

**Characteristics**			**HC**	**DC**	**AAV-Active**	**AAV-Remission**
*n*			5	11	13	6
Age, median (range), years			70 (66–72)	53 (43–87)	73 (40–85)	57 (41–70)
Male/Female			3/2	5/6	6/7	4/2
ANCA status, *n* (%)	Anti-MPO		0	0	9 (69)	3 (50)
	Anti-PR3		0	0	4 (31)	3 (50)
Diagnosis, *n* (median disease duration at sampling, month)	GPA		0	0	4 (0)	3 (143)
	MPA		0	0	9 (0)	3 (35.2)
BVAS, median (range)			N/A	N/A	16 (3–25)	0
CRP (mg/dL), median (IQR)			N/A	9 (3–26)	24 (4–60)	6 (1.8–14)
Creatinine (μmol/L), mean (SEM)			N/A	187 (63)	253 (69)	153 (52)
eGFR (mL/min), mean (SEM)			N/A	57.1 (8.3)	17.0 (7.9)	36.0 (6.9)
Immunosuppression treatment, *n* (%)	Treatment naïve		5 (100)	5 (45)	5 (38)	0
	CYC	0-6 months	0	1 (9)	1 (8)	4 (67)
		6-12 months	0	0	0	0
		>12 months	0	1 (9)	0	2 (33)
	Aza	Current	0	0	1 (8)	2 (33)
	MMF	Current	0	0	0	2 (33)
	MTX	Current	0	0	0	1 (17)
	Anti-TNF	Current	0	4 (36)	0	0
	Corticosteroids	Current	0	2 (18)	8 (62)	6 (100)
	Corticosteroids	Median duration (days, range)			1.5 (1–25)	
	Corticosteroids	Median cum dose (mg, range)			500 (60–1,780)	

^*^AAV, Anti-neutrophil cytoplasmic antibody (ANCA)-associated vasculitis; BVAS, Birmingham Vasculitis Activity Score; CRP, C-reactive protein; eGFR, Estimated glomerular filtration rate; GPA, Granulomatosis with polyangiitis; MPA, Microscopic polyangiitis; CYC, duration since cyclophosphamide exposure; Aza, Azathioprine; MMF, mycophenolate mofetil; MTX, Methotrexate.

### Density Centrifugation

Venous blood samples were collected in lithium-heparin vacutainers (Becton Dickinson, New Jersey, USA). PBMC/LDGs and NDGs were isolated by a modified Percoll (GE healthcare, Uppsala, Sweden) gradient centrifugation procedure (3, 9, 24) and stained immediately for surface markers as listed in Supplementary Table 1. Arm to stain time was <4 h in all cases. Briefly, an equal volume of 2% Dextran (Sigma-Aldrich, Missouri, USA) was added to 6–12 ml blood and inverted 20 times. Erythrocytes were left to sediment by gravity for 30 min; the supernatant was then spun at 200 g with no brake. The pellet was re-suspended in 3 ml 55% Percoll, slowly layered over 4.5 ml 65% Percoll and spun with no brake for 30 min at 1,500 g (Supplementary Figure 1). The PBMC/LDG and NDG layers were carefully removed to fresh tubes, cells were washed with PBS and the resulting cell pellets incubated with 10 ml of RBC lysis buffer (155 mM NH_4_Cl, 0.1 mM EDTA, 12 mM NaHCO_3_ pH 7.4) for 5 min. After washing, the cells were re-suspended in 1 ml of FACS buffer (2% fetal calf serum in PBS). Viability was determined using Trypan blue (Gibco) and in all cases was >90%.

### Phenotypic Analysis by Traditional Flow Cytometry

**Figure 1:**
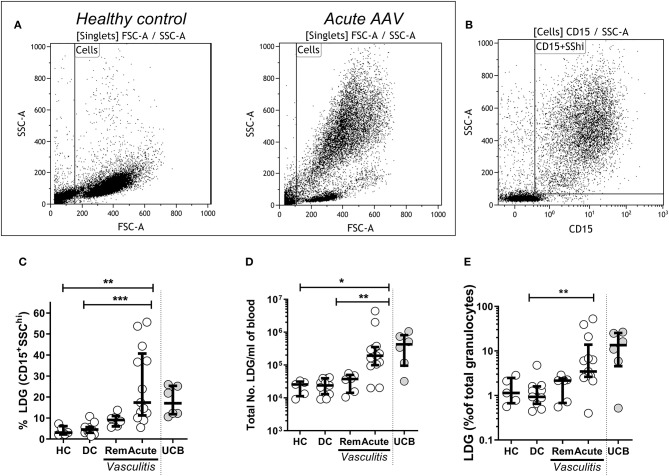
Low density granulocytes (LDGs) are elevated in patients with Acute AAV. PBMC were isolated from peripheral blood of patients with AAV by density gradient centrifugation. Representative flow cytometry dot plots are shown from healthy control PBMC and acute AAV patient PBMC. **(A)** LDGs were classified as live SSC^hi^CD15^+^ singlets (data shown from a representative patient with acute AAV). **(B)** LDGs were quantified as the percentage of PBMC in the peripheral blood of 13 acute AAV patients, 6 remission (rem), 11 disease controls (DC), and 5 age matched healthy controls (HC). Each symbol represents an individual donor. The values from 6 samples of umbilical cord blood (UCB) are shown for comparison **(C)**. The absolute numbers of LDGs/mL of blood **(D)** and percentage of total granulocytes **(E)** were also quantified. *Median with interquartile range. Kruskal-Wallis test, with post hoc analysis with Dunn's multiple comparison*. *p < 0.05, **p < 0.01, and ***p < 0.001.

The appropriate antibodies (Supplementary Table 1) were added and incubated in the dark at room temperature for 20 min. Cells were washed with PBS before being resuspended in 500 μl FACS buffer (if run immediately) or 2% paraformaldehyde (PFA) (Santa Cruz, Texas, USA) if being stored overnight at 4°C. Fluorescence minus one (FMO) controls were prepared for each fluorophore and used to define positive staining. Compensation was performed with OneComp beads (eBioscience, California, USA) stained with appropriate antibodies. A minimum of 10,000 events were collected for each sample. Cells were acquired on a FACS Canto II flow cytometer (BD, San Jose, USA) and the data were analyzed using Kaluza software (Beckman Coulter, USA). To assess the fraction and absolute cell count of LDGs in the different patient and control groups, LDGs were defined as SSC^hi^ and CD15^+^ after gating on singlets (Figure 1B). We defined the LDG fraction in three ways: (1) as a fraction of PBMCs, (2) the absolute LDG count per mL of blood, and (3) as a fraction of total granulocyte count (LDG + NDG). The latter allowed us to distinguish whether LDGs were expanded preferentially in AAV, or simply increased in proportion to total neutrophil expansion, as acute AAV is known to be associated with peripheral neutrophil leucocytosis. To further delineate the phenotypic characteristics of LDGs in comparison to paired NDGs using markers listed in Supplementary Table 1, we defined the cells as SSC^hi^CD15^+^CD14^−^ (30). Having observed differential CD16 expression we defined a CD16^−^ population (based on FMO) and CD16^+^ and CD16^int^ populations.

### Phenotypic Analysis by Imaging Flow Cytometry

After isolation from whole blood, LDGs and NDGs were immediately stained with combinations of monoclonal antibodies as detailed in Supplementary Table 1. DAPI 0.2 μg/ml (Sigma-Aldrich, Missouri, USA) was used for nuclear staining. One million cells were stained and re-suspended in 50 μl FACS buffer (2% fetal calf serum in PBS) before analysis. Images were acquired on an ImageStream X MkII imaging flow cytometer (Amnis Corporation, Seattle, WA) using INSPIRE data acquisition software (Amnis). Compensation and data analysis were performed using IDEAS 5.0 software (Amnis).

### ROS Production

ROS production was measured using the dihydrorhodamine123 (DHR123) assay as described previously (31). Briefly, 2 × 106 cells/ml from PBMCs and NDGs were suspended in separate 15 ml falcon tubes in HBH buffer (0.01% HEPES in Hank's buffered salt solution (HBSS). The cell suspension was incubated with 20 μg/ml DHR123 (Molecular Probes, D-632) and 5 μg/ml Cytochalasin B (Sigma) for 15 min at 37°C in the dark. Cells were then stimulated with 5 μg/ml anti-MPO mAb (Clone B3147M, Meridian Life sciences, Tennessee, USA) or isotype control IgG (IgG1, Origene technologies, Hanford, Germany) for 1 h at 37°C in the dark. 0.5 μg/ml Phorbol 12-myristate 13-acetate (PMA, Sigma) treated cells served as positive control. The reaction was stopped by adding 2 ml of cold HBSS (Gibco) containing 1% BSA and, after washing, the cells were stained for flow cytometric analysis as described above. Intracellular ROS production was determined by quantifying the fraction of Rhodamine123 positive cells.

### G-CSF Treatment of Humanized Mice

To assess the impact of granulocyte colony stimulating factor (G-CSF) on human CD16 granulocyte expression, we generated humanized mice as described previously (32). Briefly, NOD.Cg-*Prkdc*^*scid*^*Il2rg*^*tm*1*Wjlt*^Tg (PGK1-KITLG^*^220)441Daw/SzJ (hu-mSCF) mice were obtained from Jax (Bar Harbor, Maine, USA) and engrafted by injecting 1 × 10^5^ purified human cord blood derived CD34+ stem cells (Lonza, Slough, Berkshire, UK) into the lateral tail veins of 10–14 week-old mice ~24 h post irradiation (2.4 Gy). Following confirmation of engraftment, mice were injected subcutaneously with 50 μg pegylated filgrastim (Neulasta®, Amgen, Cambridge, UK), with repeat peripheral cell granulocyte phenotype assessed by flow cytometry 4 days later. Cells were stained with appropriate antibodies (Supplementary Table 2) after blocking with 2.5 μg/ml human BD Fc Block (clone: Fc1.3070) and 1 μg/ml mouse BD Fc Block (clone: 2.4G2). Flow cytometric analysis was performed on a CyAn ADP Analyzer (Beckman Coulter, California, USA) using Summit software (Beckman Coulter). Data were analyzed using Kaluza software. Human granulocytes were identified as hCD45^+^CD66b^+^.

### Statistical Analysis

All statistical analysis was performed using GraphPad Prism 6.0 software (GraphPad Software, San Diego, CA, USA). The LDG fraction and absolute LDG cell count were compared between groups using the Kruskal Wallis test, with comparison between individual groups using Dunn's multiple comparison test. The fraction of CD16^+^ cells between LDGs and NDGs was assessed with a Wilcoxon ranked sum test, with sub-group analysis performed using ANOVA with correction for multiple comparisons using Tukey's test. The change in CD16 expression on granulocytes in humanized mice and the variation in DHR response to stimulation were assessed with 2-way ANOVA and Sidak's multiple comparison tests. Differences between LDG CD16 subsets, and ROS production in CD10^+^ and CD10^−^ neutrophils were tested using Friedman's paired test, with *post hoc* comparison of groups using Dunn's test. The number of neutrophil lobes in CD16^+^ and CD16^int/−^ cells was compared using the Chi square test and the correlation between CD16 and CD10 expression using Spearman correlation.

## Results

### Low Density Granulocytes Are Expanded in Patients With Acute AAV

To determine whether LDGs were elevated in acute AAV, PBMC were isolated from peripheral blood of AAV patients and healthy controls (HC) by density gradient centrifugation. LDGs were initially identified as a population of high side scatter cells in acute AAV that was not present in HC (Figure 1A). These cells were further defined by their expression of CD15 (Figure 1B). We found that the LDG fraction (17.4%, IQR 11.2–40.7) and the absolute number of LDG /ml of blood (1.9 × 10^5^/ mL, IQR 1.0–3.5 × 10^5^) were significantly increased in acute AAV (Figures 1C,D). For comparison, blood containing a high fraction of immature neutrophils, umbilical cord blood (UCB), contained a median LDG fraction of 17.0% (IQR 11.8–25.4, Figure 1D). The LDG fraction represents the low-density tail of the neutrophil density distribution. As acute AAV is characterized by neutrophil leucocytosis, we assessed whether the observed LDG expansion was merely in proportion to the overall granulocyte expansion. Median LDG fraction of total granulocytes in acute AAV (3.5%, IQR 2.6–13.8) was significantly higher than in DC (Figure 1E), indicating that although total granulocyte (LDG and NDG) numbers are increased in acute AAV, there is preferential expansion of the LDG fraction.

### CD16 Expression Defines Subpopulations of LDGs in AAV

**Figure 2:**
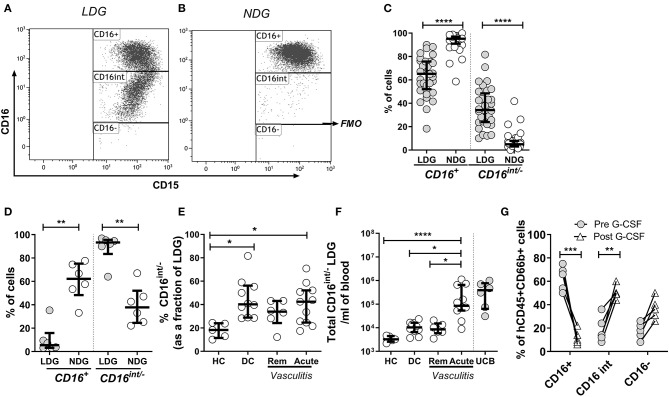
CD16 expression defines subpopulations of LDGs. Mixed leukocyte populations were separated by density centrifugation; LDGs and NDGs were classified as SSC^hi^CD15^+^CD14^−^ singlets in the low density (PBMC) and high-density layers, respectively. LDGs and NDGs were categorized as CD16^+^, CD16^int^ and CD16^−^ (FMO, fluorescence minus one), with a representative LDG sample illustrated in **(A)**. A representative NDG sample is shown in **(B)**. Most NDGs were CD16^+^, whereas approximately one third of LDGs were CD16^int/−^ **(C)** *Wilcoxon ranked sum test*. For comparison, virtually all LDGs in UCB were CD16^int/−^ **(D)**. The percentage **(E)** and absolute number **(F)** of CD16^int/−^ cells in the LDG fraction was compared across disease phenotypes. *ANOVA with post hoc Tukey's multiple comparison test*, *p < 0.05, **p < 0.01, ***p < 0.001, ****p < 0.0001. Administration of G-CSF to mice (*n* = 5) with a humanized immune system caused dramatic reduction in peripheral blood CD16^+^ neutrophils, with an associated increase in CD16^int/−^ neutrophils. *2-way ANOVA and Sidak's multiple comparison test* **p < 0.01, ***p < 0.001 **(G)**.

CD16 (Fcγ receptor III) is a low affinity receptor for IgG, expressed on neutrophils in a glycosylphosphatidyl inositol (GPI) linked form. It appears late during neutrophilic maturation. It is faintly expressed on metamyelocytes while banded and segmented stages of neutrophilic development show the highest expression (33–35). Therefore, to investigate the sub-populations within the LDG fraction CD16 expression was quantified by flow cytometric analysis. LDGs and NDGs (classified as SSC^hi^CD15^+^CD14^−^ singlets in the low density (PBMC) and high-density layer, respectively) were categorized as CD16^+^, CD16^int^ (clearly separated from CD16^+^ cells) and CD16^−^ (defined by FMO). Representative CD16 plots from LDGs (Figure 2A) and NDGs (Figure 2B) illustrate the difference in CD16 expression. In the NDG fraction in adult patients and healthy controls, 95.2% (IQR 91.0–97.2) of neutrophils were CD16^+^ which fell to 65.2% (IQR 52.1–75.7) in the LDG fraction (Figure 2C). In comparison, only 5.4% (IQR 3.2–15.9) of LDGs from UCB were CD16^+^ (Figure 2D). Consequently, there was a significant increase in CD16^int/−^ cells in the LDG fraction (34.2%, IQR 23.8–48.4) from adult patients and healthy controls compared to NDG fraction (4.9%, IQR 3.0–7.8). We hypothesized that this LDG expansion of the CD16^int/−^ population was a non-specific feature of acute illness. We found that these cells made up a greater fraction of LDGs in DC (40.2%, IQR 28.9–56.2) and patients with acute AAV (42.5%, IQR 24.6–52.1) than HC (18.3%, IQR 11.5–24.0, Figure 2E) and absolute CD16^int/−^ cell count was markedly expanded in acute AAV (Figure 2F). This non-specific expansion of the CD16^int/−^ LDG fraction in AAV suggested that these cells may be arising as a result of acute granulopoiesis leading to an increase in the number of circulating immature neutrophils (36). To test this hypothesis, we administered G-CSF to mice with a humanized immune system; this caused a dramatic reduction of peripheral blood CD16^+^ neutrophils from 62.8 ± 4.7 to 12.0 ± 2.8%, with an associated increase in CD16^int/−^ neutrophils (Figure 2G).

### LDG Surface Immune Markers Vary According to CD16 Expression

**Figure 3:**
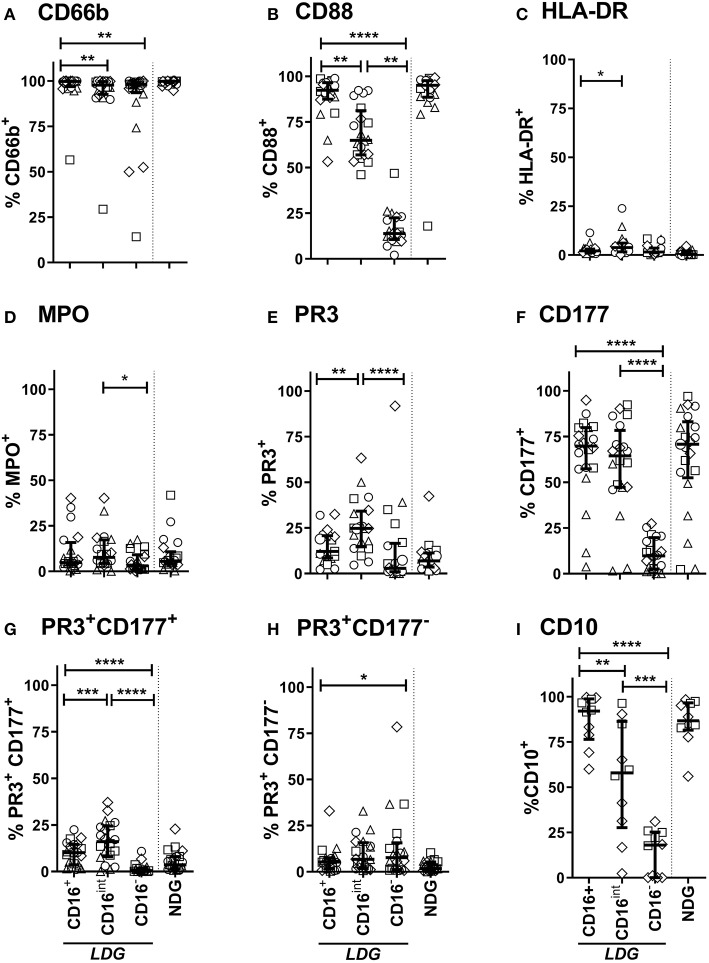
LDG Surface immune markers vary according to CD16 expression. Surface expression of CD66b **(A)**, CD88 (C5Ar) **(B)**, HLA-DR **(C)**, MPO **(D)**, PR3 **(E)**, CD177 **(F)**, PR3 CD177 co-expression **(G)**, PR3 independent of CD177 **(H)**, and CD10 **(I)** on CD16^+^, CD16^int^and CD16^−^ LDG subsets are represented. Equivalent surface expression on NDG is represented beyond the dotted line for visual comparison. Data are from Age-matched healthy control (△*n* = 5), Disease control (□ *n* = 5), Remission AAV (°*n* = 6), and Acute AAV (◇ *n* = 4) and presented as median with IQR. *Differences between LDG subsets were analyzed using Friedman's paired test, with post hoc comparison of groups using Dunn's test*. *p < 0.05, **p < 0.01, ***p < 0.001, ****p < 0.0001.

LDGs are characterized by a population of cells with high side scatter and variably low surface expression of Fc receptor CD16. To examine the phenotype of these cells in the context of AAV, we went on to define surface expression of the relevant immune markers on LDGs in detail (Table 3) and then stratified populations according to CD16 expression, with marker expression on NDGs shown alongside for comparison (Figure 3). We observed a small but statistically significant reduction in expression of the classical granulocyte marker CD66b in both CD16^int^ and CD16^−^ cells, although a high proportion of CD16^int^ and CD16^−^ LDGs (98%) were CD66b positive (Figure 3A). The alternative pathway of complement activation has recently been identified as a key pathogenic force in AAV (37). Expression of the C5a receptor (CD88), was markedly reduced on CD16^−^ cells compared to CD16^+^ (13.8%, IQR 10.6–22.5, vs. 92.3%, 87.4–96.5, respectively), with an intermediate phenotype in CD16^int^ cells (Figure 3B). Although thought to be restricted to professional antigen-presenting cells HLA-DR is present on LDGs (Figure 3C) with CD16^int^ LDGs (3.85% IQR 1.6–6.0) showing highest expression, compared to 0.3% (IQR 0.1–1.6) in NDGs. Attention was then focused on the autoantigens MPO and PR3 as they are presumed to be directly involved in cellular activation by ANCA. MPO expression was similar between LDGs and NDGs, with the highest observed in the CD16^int^ population (7.7% IQR 4.3–17.3) (Figure 3D). However, when the source of cells was stratified by disease, we observed that MPO surface expression on the CD16^−^ LDGs fraction was virtually absent in patients with AAV (1.4% IQR 0.9–4.2, Supplementary Figure 2). Expression of the autoantigen PR3 on LDGs was largely similar to NDGs, although again, a relative reduction of PR3 expression was observed in CD16- cells (Figure 3E). To explore this further, we examined expression of CD177, which is required for surface presentation of PR3 on neutrophils (38). Interestingly, CD177 was markedly reduced on CD16^−^ LDGs (9.8% IQR 2.5–19.8), compared to CD16+ LDGs (69.8%, IQR 57.3–79.9), which appeared similar to NDGs (Figure 3F). Accordingly, PR3/CD177 surface co-expression was virtually absent on CD16^−^ LDGs (0.5% IQR 0.0–1.7, Figure 3G), whereas any PR3 that was expressed on the surface of CD16^−^ cells appeared to be independent of CD177 (Figure 3H). Taken together, these findings indicate that the CD16^−^ LDG population is phenotypically distinct from other LDGs and NDGs. To address the hypothesis that these cells were immature neutrophils, we examined expression of CD10, a marker of granulocyte maturity only expressed at the segmented stage of neutrophil development. We found that CD10 expression (Figure 3I) mirrored that of CD16 and CD88. CD16^+^ LDGs (98.1% IQR 76.4–99.7) had the highest expression (similar to NDGs), while CD16^−^ LDGs (18.0% IQR 0–25.2) had the lowest.

**Table 3:** Percentage expression of phenotypic markers on LDG and NDG.

**Marker**	**LDG (Median, IQR)**	**NDG (Median, IQR)**	***p-value***
CD66b	98.7% (97.199.5)	99.8% (99.0–99.9)	0.001
CD88	71.2% (64.8–82.2)	95% (88.6–97.6)	0.003
HLA-DR	2.2% (1.1–3.4)	0.3% (0.17–1.6)	<0.0001
MPO	6.6% (3.2–11.1)	5.5% (3.9–10.8)	0.0674
PR3	14.4% (10.3–19.6)	7% (3.6–11.3)	0.0002
CD177	55.4% (45.5–70.7)	70.8% (52.4–83.1)	<0.0001
PR3+CD177+	10.4% (3.7–14.5)	3.7% (1.6–8.05)	0.0091
PR3+CD177−	5.4% (1.7–7.6)	1.7% (0.7–5.2)	0.008
CD10	75.8% (54.8–89.2)	86.7% (81.5–96.6)	0.01

### Nuclear Morphology Defines the Maturity of LDG Subsets

**Figure 4:**
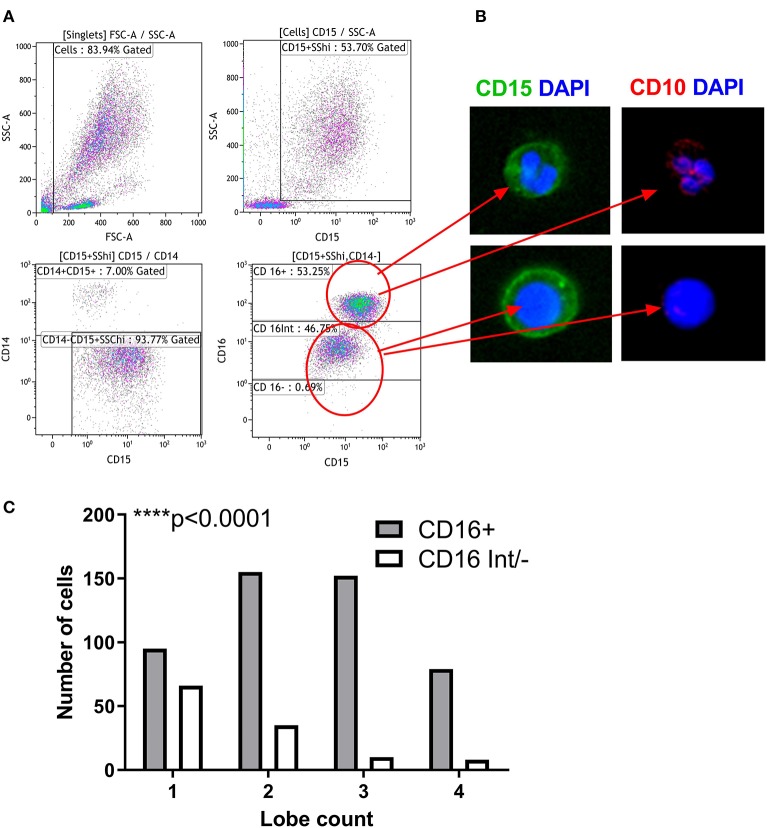
Nuclear Morphology defines the maturity of LDG subsets. Gating strategy for identification of LDG subsets by imaging flow cytometry: after gating on SSC^hi^CD15^+^CD14^−^ singlets, LDG subsets were defined as CD16^+^ and CD16^int/−^. **(A)** Representative images of LDG subsets shown. Images obtained at 60x magnification on ImageStream X MkII using DAPI nuclear stain (blue), CD15 (green), and CD10 (red). Merged images show the multilobed CD16^+^ population and the circular/kidney-shaped nuclei of the CD16^int/−^ population and the CD10 staining of the CD16^+^ subpopulations. **(B)** LDG subsets can be defined by their nuclear lobe count, a marker of granulocyte maturity. CD16^+^ LDGs have predominantly multilobed nuclei while CD16^int/−^ have mostly single lobed nuclei. *Chi square test* **(C)**. ****p < 0.0001.

About one third of the LDG population was CD16 and CD10 negative, suggesting that these cells are immature neutrophils possibly representing myeloblasts, promyelocytes, or metamyelocytes. Therefore, we used imaging flow cytometry to further characterize the LDG subsets simultaneously by both surface marker expression and nuclear morphology (Figure 4). Merged images clearly show that the CD15^+^CD16^+^ cells are multilobed while the CD15^+^CD16^int/−^ cells have circular or kidney-shaped nuclei (Figures 4A,B), with the latter combining an immature nuclear shape with absence of CD10 staining, compared to the CD15^+^CD16^+^ population, which is strongly positive for CD10. To further validate these findings, we quantified the number of nuclear lobes in all cells in the CD15^+^CD16^+^ and CD15^+^CD16^int/−^ populations using automated analysis of an imaging cytometry dataset. CD16^+^ LDGs had a median of 2 lobes whereas the CD16^int/−^ LDGs had a median of 1 lobe (Figure 4C).

### Imaging Flow Cytometry Allows Definitive Phenotyping of PBMC Cell Populations

**Figure 5:**
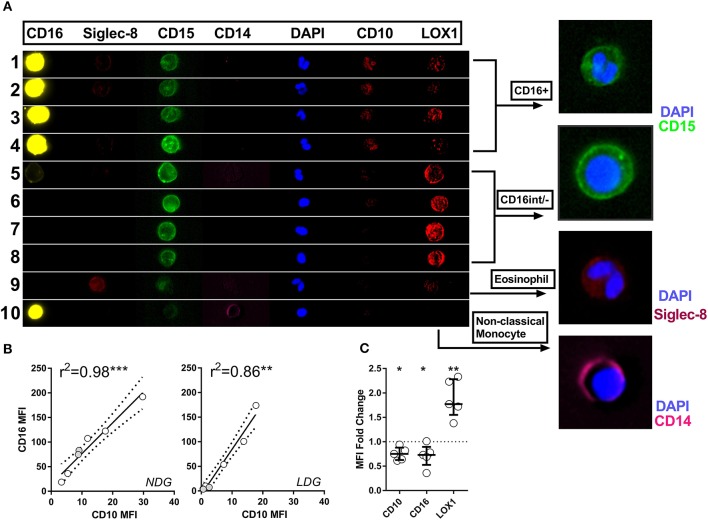
Imaging flow cytometry allows definitive phenotyping of PBMC cell populations. Representative images are shown of various cell populations from a patient with active AAV, with each row illustrating the separate channels, alongside merged images **(A)**. Rows 1–4 show CD15^+^CD16^+^ granulocytes, rows 5–8 CD16^int/−^ granulocytes, row 9 eosinophil and row 10 monocyte. CD10 expression correlates closely with CD16 expression in both LDGs and NDGs. *Spearman correlation* **(B)**. Differential CD10, CD16 and LOX-1 expression between LDG and NDG was then assessed by analyzing the fold change in MFI between the two populations. *T test* *p < 0.05, **p < 0.01, ***p < 0.001 **(C)**.

Having established that LDGs are heterogeneous based on nuclear morphology and CD16/CD10 expression, we then sought to link nuclear morphology to additional surface markers to more accurately define LDGs in the context of the PBMC population and to confirm that the CD16^−^ population are not eosinophils, which are also CD15+CD16^−^. We found that CD15^+^CD16^+^CD10^+^ cells with multi-lobed nuclei had low expression of the putative myeloid-derived suppressor cell marker LOX-1 (Figure 5A). Conversely, the corresponding CD16^int/−^ LDG population with round or bean-shaped nuclei had high LOX-1 expression. Eosinophils, with classical hinged nuclei, were clearly identified as siglec-8 positive and, like monocytes, were distinct from CD15^+^CD16^−^ LDGs (Figure 5A). CD10 and CD16 expression was highly correlated on both LDGs and NDGs (Figure 5B), suggesting that the observed low CD16 expression was due to neutrophil immaturity rather than down-regulation or shedding of this Fc receptor due to neutrophil activation or apoptosis (39, 40). The bimodal surface expression of CD16 was mirrored by CD10, whereas LOX-1 expression was unimodal (Supplementary Figure 3). LOX-1 was highly expressed on LDGs when compared to NDGs (Figure 5C); this marker may thus be useful for whole blood identification of the LDG population.

### LDGs Stimulated With Anti-MPO Antibodies Are Hypo-Responsive Compared to NDGs

**Figure 6:**
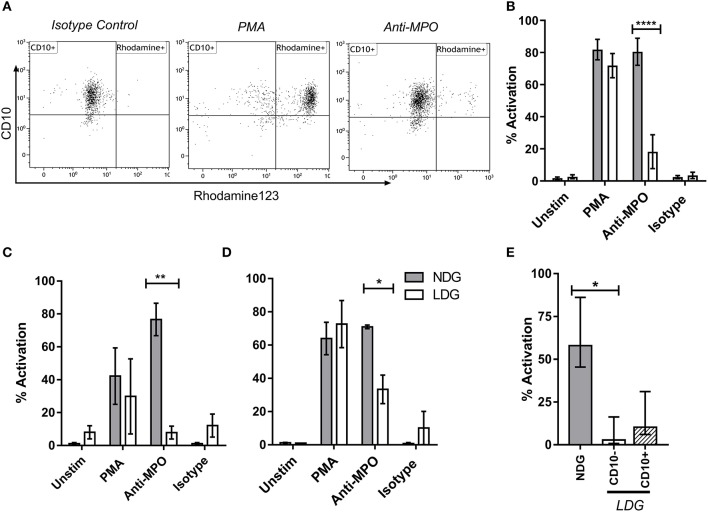
LDG stimulated with anti-MPO display decreased ROS production compared to NDG. LDG and NDG samples were stimulated with isotype control, anti-MPO antibodies, and with PMA, after loading with DHR123. ROS production was quantified as the % of Rhodamine123^+^ cells. Representative dot plots are shown demonstrating the % rhodamine123^+^ LDGs from a patient with active AAV following exposure to isotype control, PMA and anti-MPO **(A)**. ROS production by LDGs and NDGs from Acute AAV patients **(B)**, healthy controls **(C)**, and umbilical cord blood **(D)**, treated with DHR123 alone (unstim), or with DHR123 plus PMA, anti-MPO, or isotype control is shown. As ROS production was reduced in LDGs following anti-MPO stimulation, we tested whether this effect was restricted to the CD10^−^ subset **(E)**, *Friedman test with Dunn's multiple comparison test, n* = 5. *Differences between LDG and NDG response to stimulus were analyzed using 2-way ANOVA with Sidak's multiple comparison test, n* = 3 *healthy control*; *n* = 5 *Acute AAV, n* = 2 *Cord blood* *p < 0.05, **p < 0.01, ****p < 0.0001.

The production of reactive oxygen species (ROS) by neutrophils in response to anti-MPO and anti-PR3 antibodies is a well-defined functional readout of relevance to AAV (41). Dihydro-rhodamine123 is a non-fluorescent molecule which gets converted to a fluorescent molecule, rhodamine123 in the presence of ROS. As expected, NDGs produced high levels of ROS, as determined by conversion of di-hydro rhodamine to rhodamine, when stimulated with anti-MPO antibodies. However, unexpectedly, LDGs responded relatively poorly to this stimulus, despite having a good response to PMA (Figures 6A,B). We confirmed that LDGs from different clinical settings were relatively hypo-responsive to anti-MPO antibodies using healthy control and umbilical cord blood (Figures 6C,D). To test whether this response correlated with neutrophil maturity, we stratified ROS production in the LDG fraction by CD10 expression. CD10^−^ cells were completely unresponsive to anti-MPO antibodies, while CD10^+^ cells displayed an intermediate response (Figure 6E). When autoantigen surface availability was separated by CD16 expression and disease status, CD16^−^ cells from patients with AAV lacked surface MPO (Supplementary Figure 2). However, this was not observed in control cells, so cannot fully explain the lack of response in CD10^−^ cells.

## Discussion

Low density granulocytes that appear in the PBMC layer of peripheral leukocytes are recognized in diseases ranging from cancer to sepsis and autoimmunity. However, no definitive surface or functional markers for LDGs have been defined so the literature pertaining to these cells is inconsistent. We have studied LDGs in the severe autoimmune condition AAV, identifying a clear expansion in active AAV. These LDGs were phenotypically characterized by two broad cell types: CD16^+^/CD10^+^ LDGs that shared many characteristics of NDGs, and CD16^int/−^/CD10^−^ that displayed features consistent with immature neutrophils. Although other LDG work in autoimmune disease settings has classified LDGs as pro-inflammatory, mainly attributing to their ability to undergo NETosis rapidly, their response to autoantibodies hasn't been examined. Using a disease-specific ROS production assay we have shown that LDGs are unresponsive to anti-MPO stimulation, thus suggesting that LDGs unlike NDGs do not contribute to vascular damage via ROS production. Our findings suggest that the LDGs in AAV are heterogeneous, comprise a significant fraction of immature granulocytes and are unresponsive to autoantibody stimulation despite expressing MPO.

Recently, due to a surge in studies suggesting neutrophil plasticity, the concept of neutrophils as terminally differentiated innate immune cells has been brought into question and key immunomodulatory roles have been ascribed to them. Several gene expression profiling studies in AAV identified granulocyte signatures in PBMC fractions isolated by density gradient (42, 43). The neutrophil related gene expression in AAV overlapped with LDG gene expression identified in lupus and was associated with disease activity and response to treatment (44). Additionally, granulocyte transcripts detected in the blood of patients with AAV were preferentially observed in the PBMC layer, with changes in this expression correlating with subsequent relapse risk (8). It is possible that expansion of the LDG population during emergency granulopoiesis in the setting of acute disease accounts for this granulocyte signal, with transcriptionally active myelocytes and metamyelocytes exiting the bone marrow in response to G-CSF, which is known to be elevated in active AAV (45). Interestingly, in the autoimmune disease systemic lupus erythematosus, the principal upregulated genes in LDGs include serine proteases, bactericidal proteins, and other peptides present in azurophilic granules and involved in neutrophil regulation of inflammatory responses (23). These findings also suggest an immature LDG phenotype in this condition, as transcription of neutrophil serine proteases is greatest at the promyelocytic stage of neutrophil differentiation and is down regulated as neutrophils mature (46). Interestingly, a recent study utilized large scale bioinformatics approach that combined gene expression data and clinical measurements in SLE, found a core signature of 10 granulopoeisis-related genes in LDGs (47).

The accumulation of relatively immature and pathologically activated granulocytic MDSCs with potent immunosuppressive activity is well-recognized in cancer and linked to poor clinical outcome (20, 48). These have also been identified in the blood of patients with sepsis (14), cancer (26, 49), HIV (15), graft vs. host disease (50), and in pregnant women (51). Interestingly, the expression of CD10 correlates with T-cell suppression, with CD10^−^ LDGs causing T cell activation (24). An obvious question that arises is how the LDG population observed in studies in autoimmune disease relates to these granulocytic MDSCs. Attempts to answer this question have been hampered by a lack of consensus on immunophenotypic definition of these low-density cell populations (Table 1). Reliance upon density centrifugation to identify LDGs introduces a difficult to control variable and speaks to an urgent requirement for whole blood staining mechanisms that would allow for a concerted comparison of these cells across various diseases.

Table 1 compares in detail the characteristics of LDGs in various pathological conditions. LDGs from SLE patients have a pro-inflammatory phenotype. They secrete increased levels of type 1 interferon, TNF α and IFN-γ but show impaired phagocytic potential (3). A recent study found that SLE LDGs display an activated phenotype, exert proinflammatory effects on T cells and do not exhibit MDSC function (52). On the other hand, tumor associated neutrophils (TANs) are divided into two subgroups with anti-tumor (N1) or pro-tumor (N2) activity (53). A recent study shows that cancer-cell-derived G-CSF is necessary, but not sufficient, to mobilize immature low-density neutrophils (LDNs) that promote liver metastasis. In contrast, mature high-density neutrophils (HDNs) inhibit the formation of liver metastases (54). Interestingly, in multiple sclerosis (MS) the use of G-CSF to promote the recruitment and activation of neutrophils can exacerbate symptoms (55). Further work is necessary to determine the role of neutrophil subsets in different pathological settings, which is inhibited by the lack of standardization of nomenclature and classification of LDGs in these different fields.

Our observation of a lack of response of LDGs to anti-MPO stimulation casts doubt on their role as an active driver of vascular inflammation. It is conceivable that these cells are released through the action of G-CSF in the context of acute inflammation as part of a counter-regulatory homeostatic mechanism that helps to bring the immune system back to a resting state. However, given the presence of the autoantigens MPO and PR3 in this cell fraction and in immature neutrophils (56), it may also be reasonable to attribute to them a role in driving autoimmunity to these proteins. Indeed, G-CSF has been found to prime neutrophils to respond to ANCA stimulation and pre-treatment of a mouse model of MPO-AAV with G-CSF greatly exacerbates disease (45). Thus, the question as to whether LDGs arise after the onset of acute vasculitis, in response to systemic inflammation signals, or whether they act as drivers or initiators of endothelial injury, remains unanswered. Our data support the concept that they follow rather than initiate acute vasculitis, but this could only be addressed using *in-vivo* models or detailed study of relapsing patients.

## Data Availability Statement

The datasets generated for this study are available on request to the corresponding author.

## Ethics Statement

The studies involving human participants were reviewed and approved by St. Vincent's Hospital Research ethics committee, Tallaght Hospital Research ethics committee, St. James' Hospital Research ethics committee, and Beaumont Hospital Research ethics committee. The patients/participants provided their written informed consent to participate in this study.

## Author Contributions

ML, AC, GB, and EM designed and managed the project, and collated and analyzed results. AU, AC, and AD performed *in vitro* and *in vivo* experiments and analyzed data. JC and BM analyzed imaging cytometry data. KB and SD provided UCB samples. KH and JH derived clinical phenotype data. AF and UF provided rheumatoid arthritis data and samples. SM, SC, and ADB recruited patients and collected samples. All authors contributed to manuscript preparation. RL facilitated recruitment and sample collection.

### Conflict of Interest

The authors declare that the research was conducted in the absence of any commercial or financial relationships that could be construed as a potential conflict of interest.

## Supplementary Material

Supplementary Figure 1 Schematic Diagram depicting the difference between Standard, modified Percoll, and lymphoprep isolations. LDGs co-localize with PBMC layer at a density of <1.077 g/ml during density gradient preparations. Schematic representation of PBMC/LDG, neutrophil and RBC isolation from whole blood using three density (55, 65, and 85%) standard Percoll method **(A)**. PBMC/LDG and neutrophil isolation using two density (55 and 65%) modified Percoll density gradient separation **(B)** and PBMC/LDG and neutrophil isolation using 2 density Lymphoprep density gradient separation.

Supplementary Figure 2 CD16- LDG from patients with active AAV lack surface expression of the autoantigen MPO. Surface expression of MPO on CD16 subsets of LDG were analyzed for healthy control (n = 5) **(A)**, disease control (*n* = 5) **(B)** and AAV patients (*n* = 10) **(C)**. MPO surface expression was increased on CD16^+^ LDG in AAV patients, compared to healthy and disease control. Although, no differences were observed in MPO expression within CD16 subsets in healthy and disease controls, CD16^−^ LDG from AAV patients had very low MPO expression compared to CD16^+^ and CD16^int^, groups were compared using repeated-measures one-way ANOVA, ^*^*p* < 0.05.

Supplementary Figure 3 CD16 and CD10, but not LOX-1, have a bimodal distribution in LDG. Histogram overlays of CD15^+^SSC^hi^CD14^−^ singlets in LDG (dark gray) and NDG (light gray) preparations are displayed from a representative acute AAV patient sample showing surface expression of CD16 **(A)**, CD10 **(B)**, and LOX-1 **(C)**.

Supplementary Table 1 Panel of antibodies used in flow cytometry experiments.

Supplementary Table 2 Panel of antibodies used in G-CSF treated mice flow cytometric experiments.
