# Supplementary figures and images for: Low Density Granulocytes in ANCA Vasculitis Are Heterogenous and Hypo-Responsive to Anti-Myeloperoxidase Antibodies
Source: Front Immunol. 2019 Nov 7;10:2603. doi: 10.3389/fimmu.2019.02603 (PMC6856659; doi:10.3389/fimmu.2019.02603)

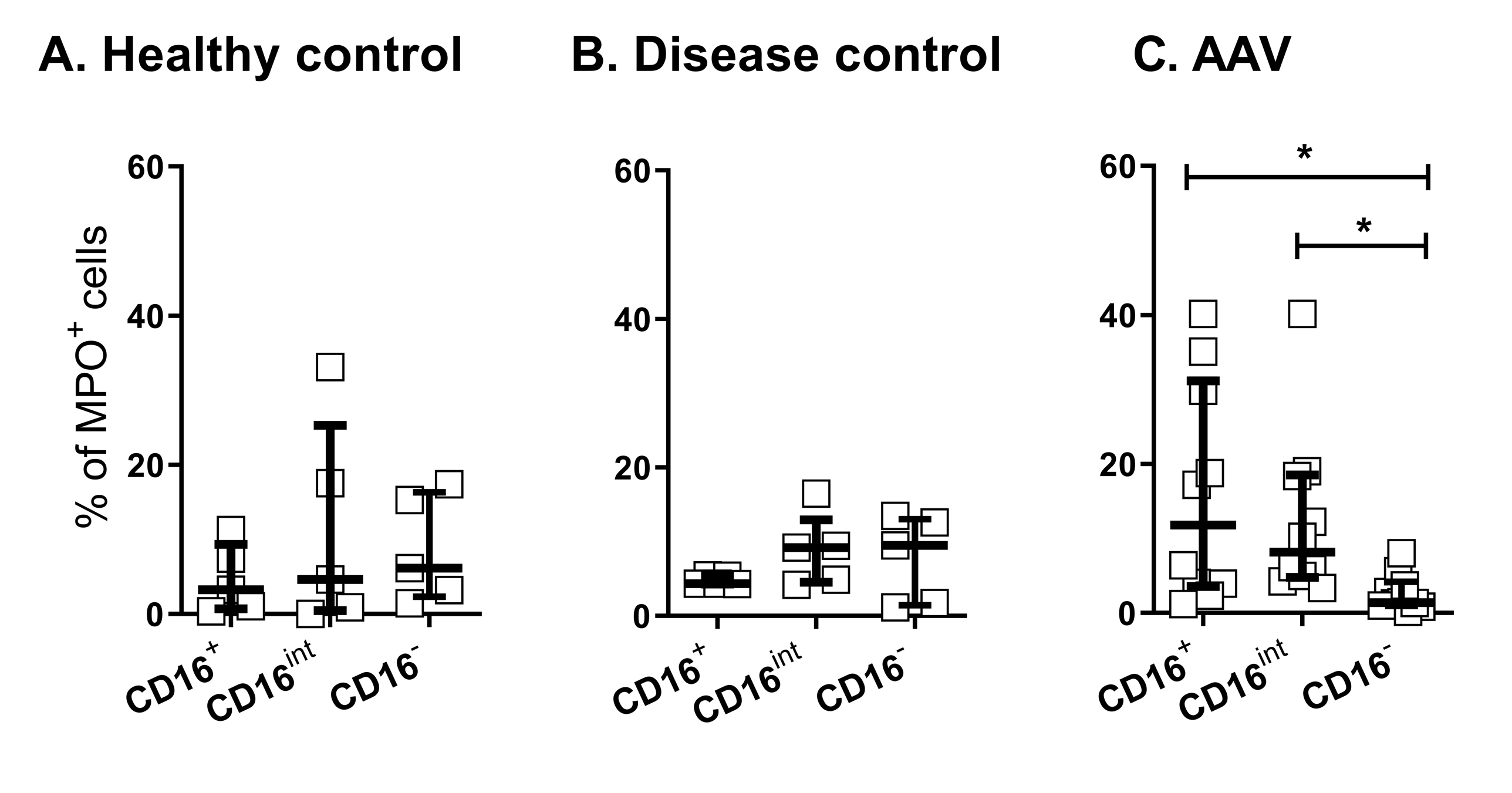

Supplement: Supplementary Figure 2 — CD16- LDG from patients with active AAV lack surface expression of the autoantigen MPO. Surface expression of MPO on CD16 subsets of LDG were analyzed for healthy control (n = 5) (A), disease control (n = 5) (B) and AAV patients (n = 10) (C). MPO surface expression was increased on CD16+ LDG in AAV patients, compared to healthy and disease control. Although, no differences were observed in MPO expression within CD16 subsets in healthy and disease controls, CD16− LDG from AAV patients had very low MPO expression compared to CD16+ and CD16int, groups were compared using repeated-measures one-way ANOVA, *p < 0.05. [file Image_2.jpg]

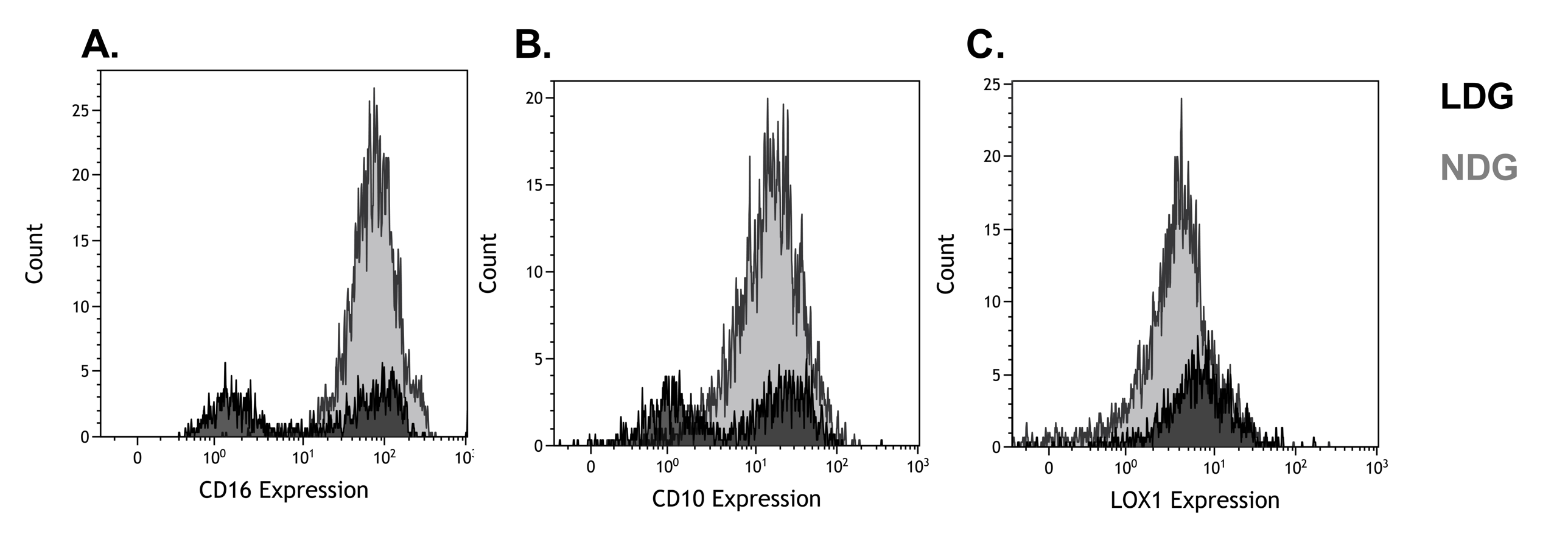

Supplement: Supplementary Figure 3 — CD16 and CD10, but not LOX-1, have a bimodal distribution in LDG. Histogram overlays of CD15+SSChiCD14− singlets in LDG (dark gray) and NDG (light gray) preparations are displayed from a representative acute AAV patient sample showing surface expression of CD16 (A), CD10 (B), and LOX-1 (C). [file Image_3.jpg]
