# Supplementary material for: Low Density Granulocytes in ANCA Vasculitis Are Heterogenous and Hypo-Responsive to Anti-Myeloperoxidase Antibodies
Source: Front Immunol. 2019 Nov 7;10:2603. doi: 10.3389/fimmu.2019.02603 (PMC6856659; doi:10.3389/fimmu.2019.02603)
Supplement: Supplementary Table 1 — Panel of antibodies used in flow cytometry experiments. [file Table_1.DOCX]

**Supplementary Table 1. Panel of antibodies used in flow cytometry experiments**

| **Antibody** | **Fluorophore** | **Clone** | **Company** |
| --- | --- | --- | --- |
| CD14 | PE-Vio770 | TÜK4 | Miltenyi Biotech |
| CD15 | PerCP/Cy5.5 | W6D3 | Biolegend |
| CD16 | AlexaFluor-488 | 368 | Biolegend |
| CD10 | BV 421 | HI 10a | Biolegend |
| Lox-1 | APC | 15C4 | Biolegend |
| Siglec-8 | PE-Dazzle | 7C9 | Biolegend |
| PR3 | FITC | W6M2 | Abcam |
| CD177 | PE | MEM-166 | Molecular Probes |
| HLA-DR | APC | L243 | Biolegend |
| CD88 | APC | S5/1 | Biolegend |
| CD66b | FITC | G10F5 | Biolegend |
| MPO | PE | 2C7 | Bio-Rad |
