# Supplementary material for: Low Density Granulocytes in ANCA Vasculitis Are Heterogenous and Hypo-Responsive to Anti-Myeloperoxidase Antibodies
Source: Front Immunol. 2019 Nov 7;10:2603. doi: 10.3389/fimmu.2019.02603 (PMC6856659; doi:10.3389/fimmu.2019.02603)
Supplement: Supplementary Table 2 — Panel of antibodies used in G-CSF treated mice flow cytometric experiments. [file Table_2.DOCX]

**Supplementary Table 2. Panel of antibodies used in G-CSF treated mice flow cytometric experiments.**

| **Antibody** | **Fluorophore** | **Clone** | **Company** |
| --- | --- | --- | --- |
| Anti-mouse CD45 | V450 | 30-F11 | Becton Dickinson, Oxford, UK |
| Anti-human CD45 | FITC | HI30 | Becton Dickinson, Oxford, UK |
| Anti-human CD66b | PE | G10F5 | Becton Dickinson, Oxford, UK |
| Anti-human CD16 | APC | 3G8 | Becton Dickinson, Oxford, UK |
